# Beyond the Microscale: Early Nanoscale Changes in Experimental Resin Composites Exposed to Acidic and Alkaline Environments

**DOI:** 10.3390/polym18212755

**Published:** 2026-11-05

**Authors:** Farah Elfakhri, Giles McCracken, Matthew German, Rawan N. AlKahtani

**Affiliations:** 1 Department of Conservative Dentistry, Faculty of Dentistry, University of Benghazi, Benghazi P.O. Box 1308, Libya; farah.elfakhri@uob.edu.ly; 2 School of Dental Sciences, Newcastle University, Framlington Place, Newcastle-upon-Tyne NE2 4BW, UK; 3 Department of Clinical Dental Sciences, College of Dentistry, Princess Nourah bint Abdulrahman University, P.O. Box 84428, Riyadh 11671, Saudi Arabia

**Keywords:** resin composite, BisGMA, TEGDMA, chemical degradation, pH, atomic force microscopy, Young’s modulus

## Abstract

The study investigates the effects of acidic, neutral, and alkaline environments on the micro- and nanoscale properties of experimental BisGMA/TEGDMA resin composites with varied filler loading. Experimental composites based on BisGMA:TEGDMA at 75:25 wt% with three silica-glass filler loadings (75, 40, and 0 wt%) were stored at 37 °C in phosphate-buffered saline (PBS; pH 7.2), citric acid (pH 3.8), or sodium hydroxide (NaOH; pH 11) for up to 28 days. Microscale changes were assessed using sorption, Vickers hardness, and scanning electron microscopy (SEM), while atomic force microscopy (AFM) was used to evaluate nanoscale surface roughness and Young’s modulus. Storage medium did not significantly affect percentage mass increase (*p* = 0.092) or Vickers hardness (*p* = 0.098). Higher filler loading was associated with lower mass increase (ρ = −0.902, *p* < 0.001) and greater hardness (r = 0.660, *p* < 0.0001). SEM revealed surface degradation, particularly at the filler–matrix interface following NaOH exposure. Nanoscale surface roughness was negatively associated with filler loading (ρ = −0.368, *p* = 0.001), with changes observed predominantly in unfilled materials after 28 days. AFM measurements demonstrated a shift towards lower Young’s modulus values after only one day of exposure to citric acid and NaOH, whereas the reduction in PBS occurred later. Acidic and alkaline exposure produced limited alterations in conventional microscale measures but more changes in surface morphology and nanoscale properties. These exploratory findings suggest that AFM-derived Young’s modulus may detect early softening following acidic and alkaline exposure, suggesting its potential utility for characterizing early changes associated with chemical ageing than conventional microscale measurements.

## 1. Introduction

Resin-based composites are continuously exposed to chemically variable conditions within the oral environment, including saliva, water, dietary acids, and other chemical agents. Penetration of fluids into the polymer network can induce water sorption, swelling, plasticisation, hydrolysis, and elution of residual components, progressively altering the physicochemical and mechanical properties of the material [1,2]. Hydrolytic processes may also compromise the silane-mediated bond between the inorganic filler and organic resin matrix, thereby contributing to interfacial degradation and loss of mechanical stability [3,4].

The susceptibility of resin composites to chemical degradation is strongly influenced by their composition. The chemistry and cross-linking characteristics of the resin matrix affect fluid uptake and polymer stability, while the amount and characteristics of the inorganic filler influence mechanical reinforcement and the relative proportion of water-susceptible organic matrix [3,5,6,7]. Increasing filler loading may therefore reduce the volume fraction of resin available for fluid uptake and improve resistance to softening, whereas variations in monomer composition, including the relative proportions of bisphenol A-glycidyl methacrylate (BisGMA) and triethylene glycol dimethacrylate (TEGDMA), can alter network structure, water sorption, and the response of the polymer to chemical ageing [8].

Environmental pH represents an additional factor governing material degradation. Acidic conditions may promote polymer plasticisation, matrix softening, and degradation at the resin–filler interface, whereas alkaline conditions can provide an accelerated hydrolytic challenge to the polymer network and silane-mediated interface [6,9,10]. Recent studies have demonstrated reductions in surface hardness and changes in surface morphology and roughness following chemical exposure of resin-based restorative materials [11,12]. However, most investigations have examined commercial composites, in which differences in resin chemistry, filler loading, filler size and composition, silanisation, and other proprietary constituents occur simultaneously [1,2,8,11]. Such compositional complexity makes it difficult to determine which material variables govern susceptibility to degradation. Systematically formulated experimental composites provide a means of isolating these effects.

The effects of chemical ageing on resin composites have also been investigated predominantly using macro- or microscale measurements, including water sorption, hardness, roughness, and microscopy [13,14,15]. Although these methods provide important information on overall material behaviour, early degradation may originate locally within the polymer matrix or at the filler–matrix interface before producing readily detectable changes at larger length scales. Atomic force microscopy (AFM) provides high-resolution surface topography and, when combined with force-mapping techniques, enables spatially resolved measurement of local mechanical properties [16,17]. Nanoscale mechanical characterisation may therefore provide a sensitive approach for identifying early or localised material changes that remain undetected by conventional microscale measurements.

Accordingly, the present study investigated three experimental BisGMA/TEGDMA composites in which filler loading were systematically varied. The materials were exposed to neutral phosphate-buffered saline (PBS; pH 7.2), citric acid (pH 3.8), or sodium hydroxide (NaOH; pH 11) for up to 28 days. Material responses were characterised at the microscale using water sorption, Vickers hardness, and scanning electron microscopy (SEM), and at the nanoscale using AFM-based surface roughness and nanomechanical measurements.

The aim of this study was to determine how acidic, neutral, and alkaline environments affect the micro- and nanoscale properties of experimental resin composites with systematically varied filler loading. Particular emphasis was placed on determining whether nanoscale mechanical characterisation may detect early chemical changes that were not apparent using conventional microscale measurements.

It was hypothesised that: (1) exposure to acidic and alkaline environments would alter the micro- and nanoscale properties of the experimental composites; (2) the magnitude of these changes would depend on filler loading; and (3) nanoscale mechanical assessment would detect early changes that were not apparent using conventional microscale measurements.

## 2. Materials and Methods

### 2.1. Experimental Composite Formulation

Three experimental resin composites were prepared by combining BisGMA:TEGDMA ratio of 75:25 with three filler loadings (75, 40, and 0 wt%). BisGMA, TEGDMA, camphorquinone (CQ), and ethyl-4-dimethylaminobenzoate (EDAB) were obtained from Sigma-Aldrich (Dorset, UK). Silanised silica-glass fillers (First Scientific Dental Materials GmbH, Elmshorn, Germany) consisted of a 1:1 mixture of 0.7 and 2.5 μm particles. 1% of CQ and EDAB were incorporated as the photoinitiator and activator, respectively, at a 1:1 ratio. The three experimental formulations are summarised in Table 1.

### 2.2. Storage Media

Three pH-based storage media were used: phosphate-buffered saline (PBS; pH 7.2), citric acid buffer (pH 3.8), and sodium hydroxide (NaOH) buffer (pH 11) (Table 2). The citric acid buffer was prepared by combining 355 mL of 0.2 M sodium phosphate dibasic with 645 mL of 0.1 M citric acid. The alkaline buffer was prepared by combining 227 mL of 0.1 M NaOH with 500 mL of 0.05 M sodium bicarbonate and diluting the mixture to a final volume of 1 L with distilled deionised water. The pH of each storage medium was verified at 37 °C using a Thermo Orion 4 Star pH meter (Thermo Fisher Scientific, Waltham, MA, USA) calibrated with standard pH 4, 7, and 9.22 buffers. The pH of each storage medium was monitored daily throughout the immersion period, including between weekly solution renewals.

The selected storage media were intended to represent contrasting chemical environments, including neutral conditions (PBS, pH 7.2), an acidic challenge (citric acid, pH 3.8), and an alkaline challenge (NaOH, pH 11). The temperature of 37 °C was selected to approximate physiological temperature, while the exposure periods of 1, 7, 14, 21, and 28 days enabled assessment of early and progressive material changes. Continuous immersion under controlled pH conditions was used as an in vitro chemical ageing model to compare material responses under standardized conditions rather than to reproduce the intermittent and fluctuating chemical exposures encountered clinically.

### 2.3. Specimen Preparation

For microscale testing, elliptical disc specimens measuring approximately 10 × 9 mm and 3 mm in thickness were prepared using a custom-made split polytetrafluoroethylene mould. The composite material was placed in the mould, covered with Mylar strips, and polymerised from both surfaces for 30 s using an LED curing unit (Bluephase, Ivoclar, Germany) at an irradiance of 1120 mW/cm^2^.

Following polymerisation, the specimens were stored for 24 h and subsequently polished under water cooling (MetaServ 2000 Twin, Buehler, Lake Bluff, IL, USA) using P600 silicon carbide paper for 60 s, followed by P800 and P1200 papers for 30 s each at 200 rpm.

### 2.4. Sorption and Mass Increase

Specimens were individually placed in glass vials and dried in a silica-gel desiccator at 37 °C until a constant mass (m_1_) was obtained. Specimen volume (*V*) was calculated as:

*V* = *πr*_1_*r*_2_*h*

where *r*_1_ and *r*_2_ are the major and minor radii, respectively, and *h* is the mean specimen thickness.

Specimens (n = 10 per formulation per solution) were individually immersed in 10 mL of PBS, citric acid, or NaOH at 37 °C for up to 28 days. Measurements were obtained after 1, 7, 14, 21, and 28 days, with the storage solutions renewed weekly. At each time point, the specimens were removed from the storage medium, blotted dry, air-dried for 15 s, and weighed (m_2_).

Sorption (S; μg/mm^3^) and percentage mass increase (MI%) were calculated as:

$$S=\frac{m2-m1}{V}$$

$$\mathrm{MI}\left(\%\right)=100(\frac{m2-m1}{m1})$$

As no post-immersion re-drying was performed, MI% represents net mass change rather than an independent measurement of water sorption or material solubility. This net change may reflect the combined effects of fluid uptake, ionic interactions, and material loss.

### 2.5. Vickers Hardness

Five specimens from each material group were evaluated at baseline. The remaining specimens were assigned to three storage media and evaluated after 1, 7, 14, 21, and 28 days of storage at 37 °C, with five independent specimens per experimental condition, yielding a total of 225 post-storage specimens. Three hardness indentations were performed per specimen.

Modified Vickers hardness (HVT) was determined using a Zwick Z2.5 universal testing machine (Zwick GmbH & Co., Ulm, Germany). Hardness values were calculated automatically by the testing software(testXpert III) using a depth-based method, based on indentation depth and the corresponding force–displacement response.

A 200 g load was applied for 20 s. Three indentations, separated by approximately 2 mm, were made on each specimen, and five randomly selected specimens were evaluated at each time point. Hardness values were calculated automatically by the testing software from the instrumented indentation measurements, using indentation depth.

### 2.6. Scanning Electron Microscopy

Surface morphology was examined using scanning electron microscopy (SEM; TESCAN VEGA-3 LMU, EM Research Services, Newcastle University). Two representative specimens from each material were examined at baseline and after 28 days of immersion in each storage medium.

Prior to examination, the specimens were dried for 48 h in a silica-containing desiccator and sputter-coated with approximately 5–10 nm of gold using a Polaron SEM coating unit. SEM micrographs were evaluated qualitatively by an assessor blinded to the specimen formulation and storage condition.

The surfaces were examined at ×10,000–15,000 magnification and qualitatively evaluated for evidence of porosity, microcracking, filler exposure or detachment, and filler–matrix interfacial degradation.

### 2.7. Atomic Force Microscopy and Nanomechanical Analysis

For nanoscale analysis, disc specimens measuring approximately 10 mm in diameter and 3 mm in thickness were prepared from each experimental formulation. The specimens were polished using the protocol described above and stored dry at room temperature for 24 h before testing.

At baseline, three randomly selected specimens from each material were analysed at three locations per specimen. Specimens assigned to chemical ageing were individually immersed in 10 mL of PBS, citric acid, or NaOH at 37 °C and evaluated after 1 and 28 days.

AFM measurements were performed using a NanoWizard 3 system (JPK Instruments, Cambridge UK) in Quantitative Imaging (QI™) mode in PBS, employing silicon nitride (Si_3_N_4_) cantilevers with a nominal tip radius of 20 nm (Bruker, Camarillo, CA, USA). Cantilever deflection sensitivity was calibrated using a stiff mica reference surface and cantilever spring constants (0.35 N/m) were calibrated using the JPK software (V8) calibration procedure based on the thermal-noise method. Specimens were immediately transferred from their respective storage solutions for AFM assessment, with measurements performed under hydrated conditions in PBS.

Three randomly selected areas, approximately 1 mm apart, were examined on each specimen. Each scan covered an area of 15 × 5 μm^2^ at a resolution of 256 × 85 pixels. The three-dimensional arithmetic mean surface roughness (Sa) was obtained from the QI™ images to quantify nanoscale surface topography. Young’s modulus was calculated from the approach portion of the AFM force–distance curves using the Hertz contact model in the JPK data-processing software, assuming a quadratic-pyramidal tip geometry. A maximum applied load of 1500 nN was used. Multiple probes of the same model were required during the experiments due to probe damage and wear.

*Interpretation of Young’s modulus distributions*

AFM-derived Young’s modulus measurements were presented as histograms and evaluated descriptively. The interpretation considered distribution shape, spread, shifts towards lower or higher modulus values, and the presence or absence of high-modulus tails. This approach was used to characterise variations in the local nanomechanical response of the heterogeneous experimental composites. Individual force-curve measurements were treated as nested observations rather than independent experimental replicates. No specimen-level inferential statistical comparisons of Young’s modulus were performed; therefore, differences between experimental conditions were interpreted descriptively.

### 2.8. Statistical Analysis

Statistical analyses were performed using SigmaPlot 13.0. Data normality and homogeneity of variance were assessed using the Shapiro–Wilk and Brown–Forsythe tests, respectively. Data are presented as medians and interquartile ranges (IQRs) where appropriate.

Sorption data were analysed using a general linear model to examine the effects of filler loading, storage medium, and storage duration, followed by Tukey’s multiple-comparison test where appropriate. The overall effect of storage medium on percentage mass increase (MI%) was assessed using a general linear model, while the Kruskal–Wallis test was employed for supplementary nonparametric comparisons.

Spearman’s rank-order correlation was used to assess associations between filler loading, storage duration, sorption, and percentage mass increase.

Vickers hardness data were analysed using a general linear model to examine the effects of filler loading, storage medium, and storage duration, followed by Tukey’s multiple-comparison test where appropriate. Spearman’s rank-order correlation was additionally used to assess associations between Vickers hardness, filler loading, and storage duration.

Surface roughness (Sa) was analysed using three-way analysis of variance (ANOVA) to examine the effects of filler loading, and storage conditions, followed by Tukey’s post-hoc test where appropriate. Within-solution comparisons were performed using the Kruskal–Wallis test when parametric assumptions were not satisfied. Spearman’s rank-order correlation was used to assess associations between surface roughness, filler loading, and storage duration.

Young’s modulus data obtained from AFM force mapping were analysed descriptively using histograms and 95% quantile confidence intervals to characterise local nanomechanical responses according to material composition, storage medium, and duration. Individual force-curve measurements were considered nested observations rather than independent experimental replicates. As no specimen-level inferential statistical analysis was performed, differences in Young’s modulus distributions between experimental conditions were interpreted descriptively.

Statistical significance was set at α = 0.05.

## 3. Results

### 3.1. Sorption and Mass Increase

Sorption data were non-normally distributed with unequal variances. Storage medium had no significant overall effect on percentage mass increase (MI%; *p* = 0.092), Although PBS exhibited the highest median MI% across all filler loadings, the relative values for citric acid and NaOH varied depending on filler loading (Table 3).

Filler loading showed a strong negative correlation with MI% (ρ = −0.902, *p* < 0.001), with highly filled composites exhibiting the lowest mass increase and unfilled materials generally exhibiting the highest.

### 3.2. Vickers Hardness

**Figure 1:**
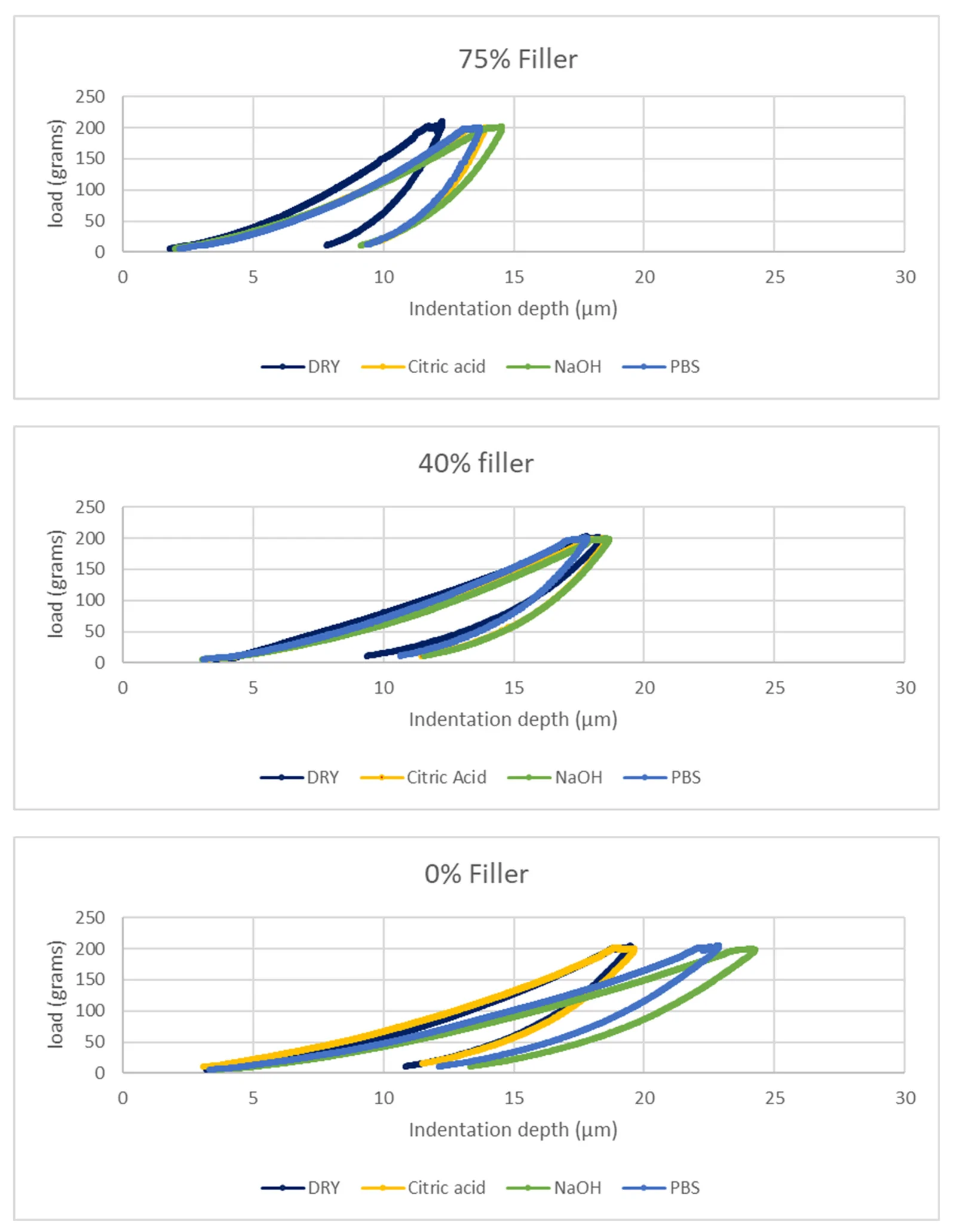
Typical load–displacement curves during the indentation of (75%BisGMA) experimental groups with three filler loadings at baseline reading (DRY) and after storage for 28 days in different solvents: PBS, citric acid, and NaOH.

Filled composites exhibited greater Vickers hardness than unfilled materials both at baseline and following chemical ageing (Table 4, Figure 1). Filler loading showed a moderate positive correlation with Vickers hardness (ρ = 0.660, *p* < 0.0001).

Storage medium had no significant overall effect on Vickers hardness (*p* = 0.098). Storage duration showed a very weak negative correlation with Vickers hardness (ρ = −0.0713, *p* = 0.287); however, this association was not statistically significant in the specimen-level analysis.

### 3.3. SEM Surface Morphology

**Figure 2:**
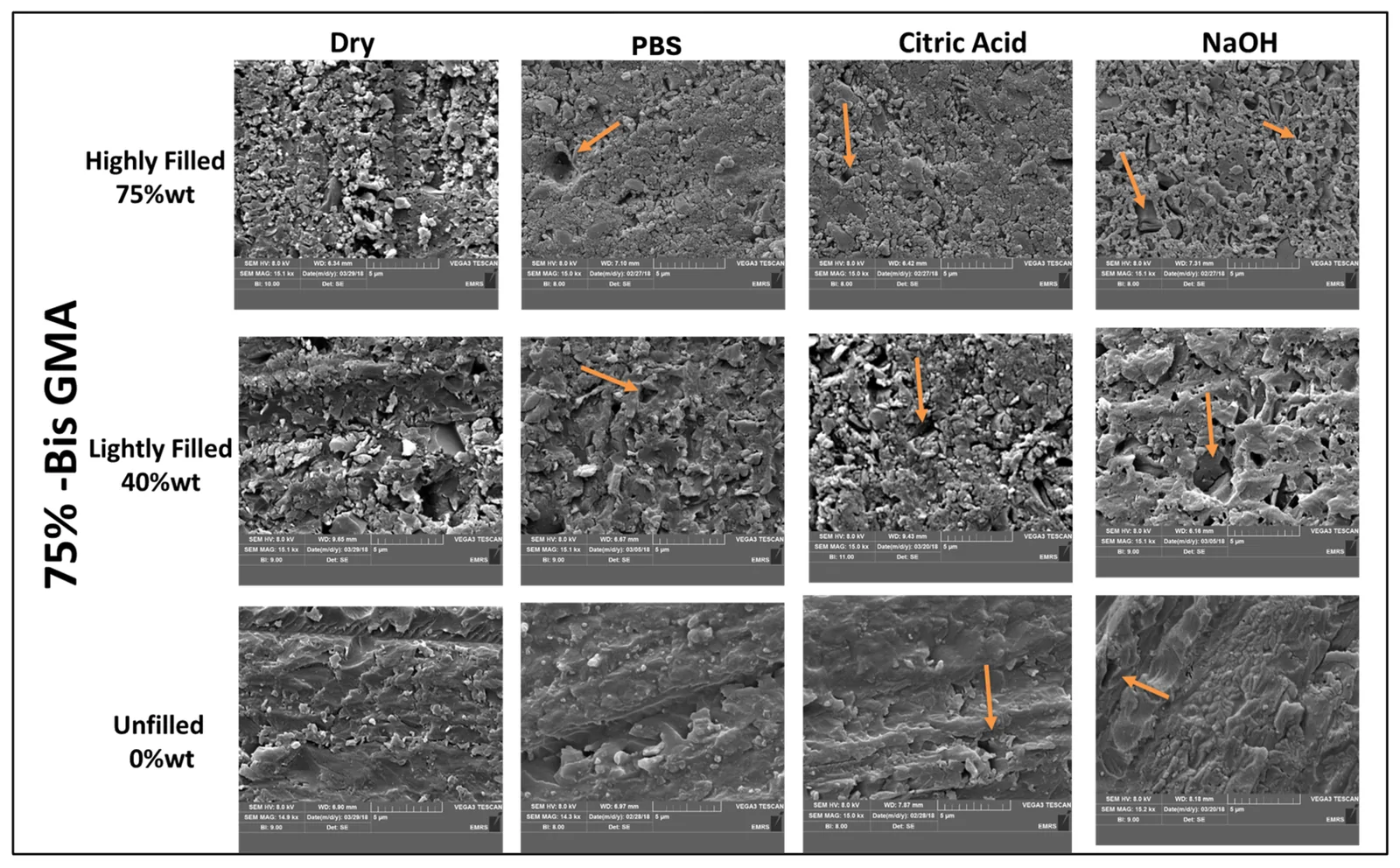
SEM micrographs of (75% BisGMA) material samples with different filler ratios before and after storage for 28 days in different solutions. Arrows showing microporosity, micro-cracks in the matrix, traces of detached fillers and interfacial debonding.

SEM examination revealed changes in surface morphology following 28 days of chemical ageing (Figure 2). Filled composites exhibited microporosity, matrix microcracking, filler exposure or detachment, and evidence of filler–matrix interfacial debonding.

Among the storage media, NaOH exposure produced the most pronounced morphological changes in filled composites, particularly at the filler–matrix interface, whereas specimens exposed to citric acid and PBS exhibited less extensive surface alterations.

In contrast, unfilled materials predominantly exhibited surface porosity and matrix microcracking, without the filler-related interfacial changes observed in filled composites.

SEM analysis was intended to provide a qualitative comparison of surface morphology between baseline and 28 days of chemical ageing rather than a quantitative or longitudinal assessment of surface degradation.

### 3.4. AFM Surface Topography

**Figure 3:**
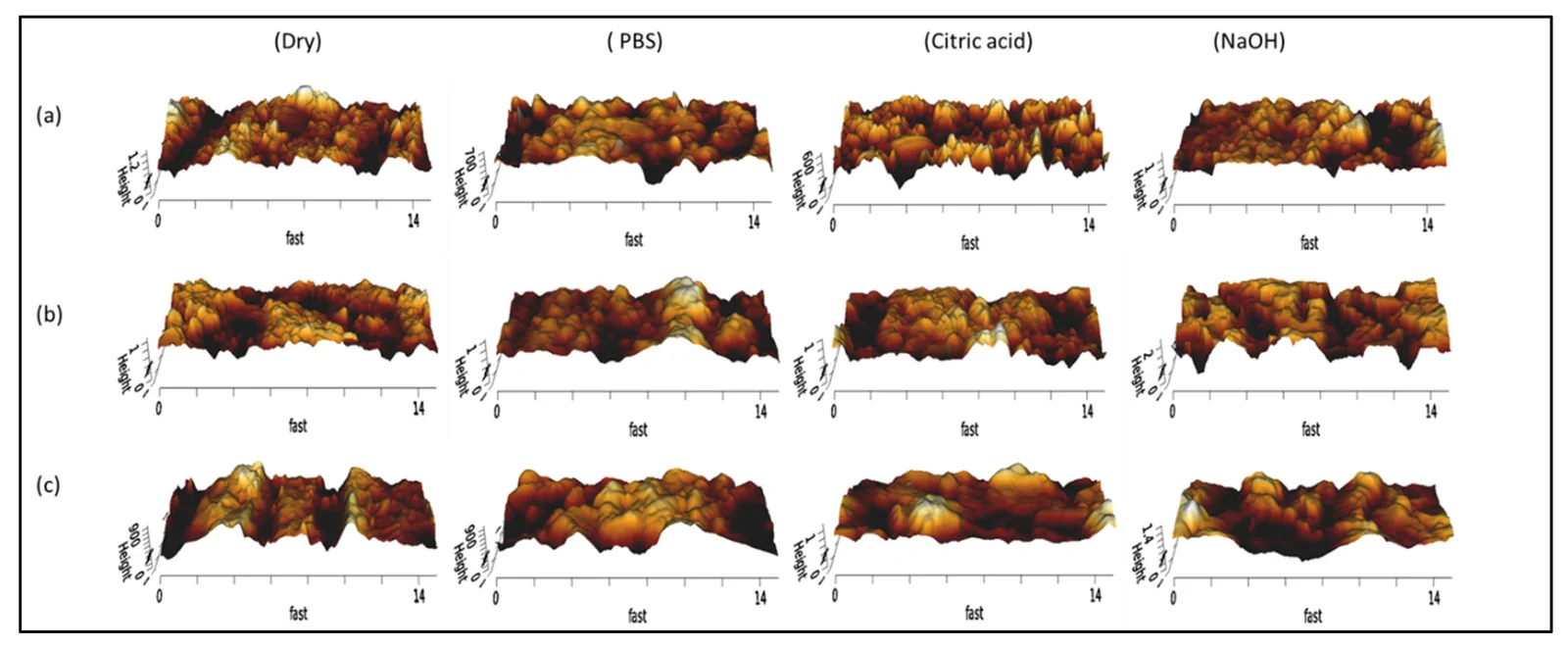
Representative 3D images for the three filler loadings—(**a**) 75%, (**b**) 40%, and (**c**) 0%—of 75% BisGMA materials in the dry state and after 28 days of storage in different pH solutions.

Baseline AFM topographic images showed generally smooth surfaces with occasional polishing scratches. The AFM topographic images revealed surface features in the 40 and 75 wt% formulations that may be associated with the presence of filler particles (Figure 3).

Qualitative examination of AFM topographic images following storage in PBS, citric acid, or NaOH revealed no major changes in surface appearance compared with baseline. However, quantitative analysis of surface roughness and nanomechanical properties identified changes that were not readily apparent from qualitative assessment of the AFM topographic images.

### 3.5. Nanoscale Surface Roughness

**Figure 4:**
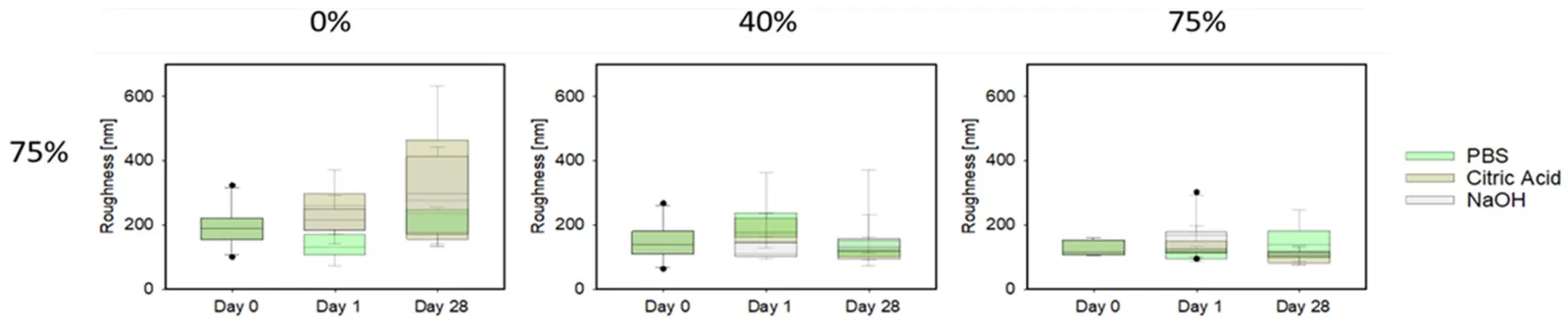
Box-and-whisker plots showing nanoscale surface roughness (Sa) of the three experimental composites at baseline (Day 0) and after 1 and 28 days of storage in different pH solutions. The central line represents the median, the box indicates the interquartile range (25th–75th percentiles), and the whiskers extend to the most extreme observations within 1.5 times the interquartile range. Values beyond the whiskers are shown as outliers.

Baseline median Sa values ranged from approximately 75.6 to 185.7 nm, with no significant differences among the three formulations (*p* = 1.00) (Table 5, Figure 4).

Following chemical ageing, changes in surface roughness were most apparent after 28 days, in the unfilled materials. However, the magnitude and direction of Sa changes varied across filler loadings, storage media, and exposure durations.

Filler loading showed a significant negative correlation with surface roughness (ρ = −0.368, *p* = 0.001), indicating lower roughness with increasing filler loading.

Day-1 roughness values were generally comparable to baseline. Across the overall dataset, storage duration showed no meaningful correlation with surface roughness (ρ = −0.011, *p* = 0.921).

### 3.6. AFM Nanomechanical Properties

**Figure 5:**
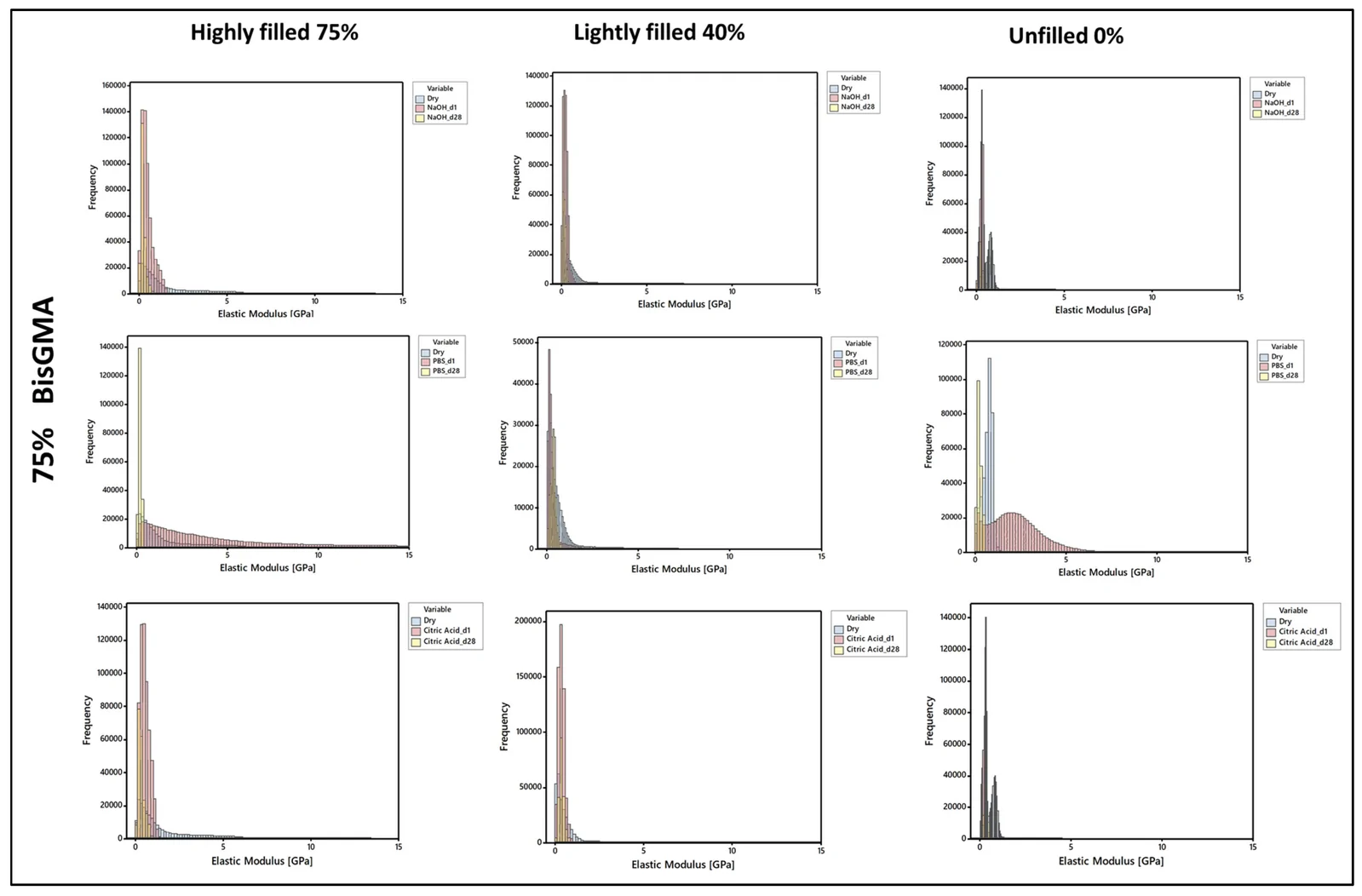
Histogram distributions of AFM-derived Young’s modulus for experimental resin composites containing 75, 40, and 0 wt% filler at baseline (Day 0) and following 1 and 28 days of storage in PBS, citric acid, and NaOH.

The distribution of AFM-derived Young’s modulus measurements showed distinct responses to the storage media (Figure 5). Following exposure to citric acid and NaOH, the distributions shifted towards lower modulus values after one day, with this pattern remaining evident at 28 days. In filled composites, the baseline distributions were generally skewed towards higher values, with a high-modulus tail that was no longer evident after one day in acidic or alkaline media. Unfilled composites showed predominantly unimodal distributions but also shifted towards lower modulus values following these exposures. PBS produced a different temporal pattern. The distributions generally shifted towards higher modulus values after one day, followed by a shift towards lower values at 28 days.

Interpretation of these findings was based on the overall histogram distributions, rather than on a single measure of central tendency. The changes in distribution shape and spread, particularly the disappearance of the high-modulus tail in filled composites, provided additional information about the local nanomechanical response following chemical exposure. The tail may reflect AFM measurements of mechanically harder, filler-rich regions; its disappearance does not, by itself, demonstrate filler dissolution or loss.

## 4. Discussion

The principal finding of this study was that the material response to chemical ageing depended on both the property measured and the scale of assessment. Exposure to PBS, citric acid, and NaOH produced relatively limited changes in conventional microscale measurements, with no significant overall effect of storage medium on percentage mass increase (MI%; *p* = 0.092) or Vickers hardness (*p* = 0.098). Nevertheless, SEM revealed morphological evidence of degradation after 28 days, while AFM identified changes in surface roughness and, most notably, an early reduction in Young’s modulus following acidic and alkaline exposure. These findings suggest that local nanomechanical alterations may develop before substantial changes become detectable using conventional microscale measurements.

### 4.1. Influence of Material Composition and Storage Environment on Microscale Properties

The absence of a significant difference in MI% among PBS, citric acid, and NaOH suggests that, within the 28-day experimental period, storage-medium pH was not the dominant determinant of overall fluid uptake. This is consistent with evidence that the effect of acidic media on fluid uptake is material-dependent and influenced by compositional factors such as filler loading, filler characteristics, and polymer-matrix chemistry [7,18,19].

In contrast, filler loading had a pronounced influence on mass increase, with MI% decreasing as filler concentration increased (ρ = −0.902, *p* < 0.001). This finding is consistent with previous studies demonstrating that increasing inorganic filler content reduces the relative proportion of polymer matrix available for fluid penetration [20,21,22]. Within the systematically controlled formulations investigated here, filler loading therefore appeared to have a greater influence on fluid uptake than the storage-medium pH.

A similar compositional effect was observed for Vickers hardness. Increasing filler loading was moderately correlated with greater hardness (ρ = 0.660, *p* < 0.0001), consistent with the reinforcing contribution of the inorganic phase to the mechanical properties of resin composites [20,21,22,23]. Storage medium, on the other hand, had no significant overall effect on Vickers hardness (*p* = 0.098). Although storage duration showed a very weak negative correlation with Vickers hardness (ρ = −0.0713, *p* = 0.287), this association was not statistically significant. The negligible magnitude of the correlation suggests that storage duration alone did not account for substantial variation in hardness. Rather, the findings indicate that although early reductions in hardness were observed in several groups, including PBS-exposed specimens, storage medium had no statistically significant overall effect on Vickers hardness. Examination of the Day-1 measurements revealed a notable reduction in median Vickers hardness in the 75 wt% filler formulation exposed to NaOH, from 63 VHN at baseline to 27 VHN after 24 h, followed by a higher median value of 48 VHN at Day 28. An early reduction was also observed in PBS-exposed unfilled specimens (29 to 16 VHN), suggesting that the initial transition from dry to hydrated conditions and associated water-induced plasticisation may have contributed to the observed hardness changes.

Previous investigations have similarly demonstrated that hardness changes during ageing are not necessarily monotonic, with increases observed following initial storage or subsequent ageing procedures [24,25]. Although differences in experimental protocols limit direct comparisons, these findings suggest that the temporal response of composite hardness may be more complex than progressive softening alone. The mechanism underlying the present observations remains uncertain, particularly given the nonsignificant overall effect of storage medium on Vickers hardness.

The limited effect of NaOH on Vickers hardness contrasts with previous reports of filler loss, interfacial deterioration, matrix cracking, and reductions in mechanical properties following alkaline degradation [26,27,28,29]. Differences in exposure severity may partly explain this discrepancy. The present study used NaOH at pH 11 and 37 °C, whereas some accelerated-degradation protocols have employed substantially more aggressive alkaline conditions, including pH values approaching 13 and temperatures of approximately 60 °C [26]. The comparatively moderate conditions used in the present study may therefore have been insufficient to produce pronounced changes in bulk hardness within 28 days.

### 4.2. Morphological and Nanoscale Surface Changes

The limited changes in MI% and Vickers hardness did not indicate an absence of structural degradation. SEM examination after 28 days revealed surface porosity, matrix microcracking, filler exposure or detachment, and evidence of filler–matrix interfacial deterioration. These changes were most apparent in NaOH-exposed filled composites, particularly at the filler–matrix interface. Such morphology is consistent with alkaline hydrolysis of the silane-mediated interface, as hydroxyl ions may promote hydrolytic cleavage of siloxane bonds and compromise coupling between the inorganic filler and polymer matrix [26,28,30]. In contrast, unfilled formulations lacked this interface and predominantly exhibited porosity and matrix microcracking.

Citric acid- and PBS-exposed specimens showed less pronounced morphological changes. Taken together, the SEM observations illustrate an important distinction between local structural degradation and bulk mechanical response: morphological evidence of deterioration may be present even when the process is not sufficiently extensive to produce a statistically detectable change in conventional hardness.

The relatively higher baseline Sa observed in the unfilled formulation may reflect differences in surface preparation and inherent polymer-matrix topography rather than filler-related effects, although baseline differences were not statistically significant. AFM surface analysis further demonstrated that the response to chemical ageing depended on the parameter examined. Filler loading was negatively correlated with nanoscale surface roughness (Sa; ρ = −0.368, *p* = 0.001), with the largest roughness changes occurring predominantly in unfilled formulations after prolonged acidic or alkaline exposure. The greater proportion of exposed polymer matrix in these materials may have increased their susceptibility to chemically induced surface alteration. This interpretation is consistent with previous observations that composite composition influences surface degradation following chemical exposure [31,32,33].

However, surface roughness did not provide a consistent marker of early degradation. Day-1 values were generally comparable to baseline, and storage duration showed no significant overall correlation with roughness (ρ = −0.011, *p* = 0.921). Thus, the present findings do not indicate that nanoscale measurements are inherently more sensitive than conventional measurements; rather, sensitivity to early degradation depended on the specific property being assessed.

### 4.3. Early Nanomechanical Changes

The most distinctive response was observed in AFM-derived Young’s modulus. Exposure to citric acid and NaOH was associated with apparent shift towards lower Young’s modulus values after only one day, and this reduction remained evident after 28 days. PBS showed a different temporal pattern, with modulus values generally increasing at Day 1 before shifting towards lower values by Day 28. This early response to acidic and alkaline exposure contrasted with the absence of a significant overall storage-medium effect on Vickers hardness.

The reduction in Young’s modulus may reflect plasticisation and hydrolytic alteration of the polymer network. Water uptake by BisGMA/TEGDMA-based polymers can affect intermolecular interactions and increase polymer-chain mobility, resulting in a reduction in mechanical properties [19,34]. Acidic environments have also been associated with swelling and plasticisation of polymer networks, whereas alkaline environments may promote hydrolytic degradation of both the polymeric phase and the silane-mediated filler–matrix interface [9,19,35,36]. These mechanisms provide possible explanations for the observed nanomechanical response, although they were not directly measured in the present study.

The distribution-level analysis provided complementary information regarding the heterogeneous local mechanical response of the experimental composites. Because these materials contain mechanically distinct resin and filler phases, a single summary value may not adequately capture changes in distribution shape or the presence of high-modulus tails. At baseline, filled composites exhibited right-skewed distributions with high-modulus tails, consistent with the presence of mechanically distinct filler-rich regions. Following exposure to citric acid and NaOH, the distributions shifted towards lower modulus values, and the high-modulus tails became less apparent. In contrast, these features remained detectable after one day of PBS exposure, with a more pronounced shift towards lower modulus values following prolonged storage.

Importantly, the disappearance of the high-modulus tail should not be interpreted as direct evidence of filler dissolution, loss, or softening. One possible explanation is that chemical exposure induced changes in the surrounding polymer matrix, including softening or swelling, which altered the local mechanical response detected by the AFM probe. Such changes may have reduced the distinction between mechanically stiffer filler-rich regions and the surrounding matrix, contributing to the observed changes in the modulus distributions. However, this interpretation remains hypothetical, as the histograms do not establish the underlying degradation mechanism. Furthermore, given the local nature of AFM measurements and the absence of specimen-level inferential statistical testing of Young’s modulus, these distributional changes should be interpreted as descriptive rather than statistically confirmed effects. The SEM evidence of matrix and interfacial deterioration after prolonged exposure is consistent with this interpretation, although the underlying mechanism cannot be established from the present measurements alone.

### 4.4. Relationship Between Microscale Hardness and Nanoscale Modulus

The contrasting responses of Vickers hardness and AFM-derived Young’s modulus are central to the interpretation of the present findings. Vickers indentation assesses the response of a comparatively larger volume of heterogeneous material and therefore reflects the combined contributions of the polymer matrix, filler particles, and filler–matrix interface [37]. Localised early degradation affecting predominantly the polymer matrix or interfacial regions may consequently have a limited effect on the overall hardness measurement.

In contrast, AFM force mapping interrogates mechanical behaviour over a much smaller spatial scale and can resolve local variations across heterogeneous material regions. Changes in the compliance of the polymer matrix or local filler–matrix environment may therefore become apparent before they are sufficiently extensive to alter conventional hardness. In the present study, this distinction was reflected by the reduction in AFM-derived Young’s modulus after only 24 h of citric acid or NaOH exposure despite the absence of a significant overall storage-medium effect on Vickers hardness.

Importantly, this finding should be attributed specifically to AFM-derived Young’s modulus rather than to nanoscale characterisation in general. Surface roughness did not demonstrate the same early and consistent response. Young’s modulus therefore may provide the more sensitive nanoscale indicator of early pH-associated mechanical alteration under the experimental conditions investigated.

The combined micro- and nanoscale findings are summarised in Table 6. While storage medium had no statistically significant overall effect on mass increase or Vickers hardness, SEM revealed morphological alterations, particularly at the filler–matrix interface following alkaline exposure. AFM-derived Young’s modulus demonstrated earlier changes following acidic and alkaline exposure, suggesting that local nanomechanical alterations may become detectable before substantial changes are apparent in conventional microscale measurements. Together, these findings highlight the complementary value of assessing chemical degradation across different structural scales.

### 4.5. Clinical Relevance, Limitations, and Future Directions

The present findings suggest that the early stages of resin-composite degradation may not be fully reflected by conventional bulk measurements. Although storage medium had no significant overall effect on mass increase or Vickers hardness, morphological deterioration and reductions in local Young’s modulus were detected following chemical ageing, with nanomechanical changes occurring as early as 24 h after acidic or alkaline exposure. Clinically, restorative composites are repeatedly exposed to fluctuations in oral pH arising from dietary intake and other environmental challenges. The detection of early local softening under such conditions suggests that a restoration may undergo subclinical material changes before measurable deterioration in conventional bulk properties becomes evident. Over repeated or prolonged exposure, these local alterations could potentially contribute to progressive matrix and filler–matrix interfacial degradation. The findings therefore highlight the importance of considering resistance to chemical ageing, in addition to initial bulk mechanical properties, when evaluating the long-term performance of resin-based restorative materials.

Additionally, findings should be interpreted within the context of a controlled in vitro ageing model. Continuous exposure to solutions of fixed pH does not reproduce the dynamic oral environment, where pH fluctuates and materials are simultaneously exposed to saliva, temperature changes, dietary components, oral hygiene products, and mechanical loading. In addition, the simplified experimental BisGMA/TEGDMA formulations were designed to isolate the effects of filler loading and therefore do not represent the compositional complexity of contemporary commercial composites. The observed association between filler loading and surface roughness may partly reflect differences in baseline surface characteristics among formulations and should not be interpreted as a direct measure of ageing-induced roughness changes. Furthermore, the limited number of specimens examined by AFM (n = 3 per group) and the absence of a time-matched dry control constrain the interpretation of the observed nanomechanical changes.

AFM measurements are also highly localised and sensitive to surface characteristics and probe–sample interactions; consequently, the measured modulus distributions represent local surface nanomechanical behaviour rather than bulk elastic properties. The unexpectedly low baseline modulus values may also reflect uncertainties associated with cantilever calibration, probe characteristics, and contact-model assumptions, limiting confidence in their absolute accuracy.

Furthermore, SEM analysis was limited to qualitative observations at baseline and after 28 days of exposure. Consequently, the temporal progression and extent of surface deterioration could not be quantified. Additional SEM assessments at intermediate time points, quantitative image analysis, and complementary energy-dispersive X-ray spectroscopy (EDS) would provide further insight into morphological progression and potential changes in local elemental composition. Additionally, drying specimens for 48 h before SEM examination may have induced or accentuated morphological artifacts, including matrix shrinkage, microcracking, and interfacial gaps. Therefore, the potential contribution of drying-related artifacts to the observed surface alterations cannot be excluded. The early changes in AFM-derived Young’s modulus suggest that local nanomechanical measurements may detect material responses that are not readily apparent through conventional bulk measurements. However, these changes cannot be attributed exclusively to chemical degradation, as the measured modulus may also be influenced by hydration, surface topography, local material heterogeneity, probe geometry, and contact conditions. Although probe type remained consistent across experimental groups, replacement of individual probes may have introduced measurement variability. Furthermore, the relatively high contact load (1500 nN) may have influenced the local mechanical response, and the absence of independent post-indentation surface assessment prevents exclusion of measurement-induced deformation.

In the absence of direct chemical characterisation, the underlying mechanisms remain uncertain. Additionally, Young’s modulus was assessed using descriptive distributions of nested force-map measurements without specimen-level inferential testing. Consequently, the observed distributional shifts should be interpreted as exploratory rather than statistically confirmed effects of storage conditions. Degree of conversion was not assessed in the present study; therefore, potential variations in polymerisation associated with filler loading and specimen thickness cannot be excluded.

Further studies should determine whether the early nanomechanical changes identified here translate into progressive deterioration under conditions that more closely simulate clinical service. This should include artificial or human saliva, thermocycling, and combined chemical–mechanical ageing over longer periods. Evaluation of contemporary commercial composites with different resin chemistries, filler systems, and coupling technologies would establish whether the observed response extends beyond simplified BisGMA/TEGDMA formulations. Longitudinal studies combining AFM force mapping with conventional mechanical testing and complementary chemical characterisation, such as FTIR or Raman spectroscopy and analysis of released degradation products, would help investigate the mechanisms underlying the early reduction in Young’s modulus and determine whether these nanoscale changes can predict subsequent bulk degradation or clinically relevant material deterioration.

## 5. Conclusions

Within the limitations of this experimental study, the effects of chemical ageing were dependent on material composition and the scale of assessment. Storage environment had no significant overall effect on percentage mass increase or Vickers hardness, whereas filler loading strongly influenced fluid uptake, hardness, and nanoscale surface roughness. SEM nevertheless revealed morphological degradation, particularly at the filler–matrix interface following NaOH exposure. Most notably, AFM-derived Young’s modulus showed early reductions following citric acid and NaOH exposure that were not apparent from conventional microscale measurements.

These findings partially support the first hypothesis and support the influence of filler loading proposed in the second hypothesis. AFM-derived Young’s modulus distributions suggested early local nanomechanical changes following acidic and alkaline exposure. These descriptive findings provide preliminary support for the third hypothesis, although confirmation through specimen-level statistical analysis is required.

Overall, AFM-derived Young’s modulus may provide a sensitive indicator of early changes associated with chemical ageing in experimental resin composites and may complement conventional methods in evaluating material stability. These AFM-derived modulus findings are exploratory and require further validation before broader conclusions can be drawn.

## Figures and Tables

**Table 1:** Composition of the experimental resin composites. BisGMA, bisphenol A-glycidyl methacrylate; TEGDMA, triethylene glycol dimethacrylate. Fillers consisted of a 1:1 mixture of 0.7 and 2.5 μm silanised silica-glass particles.

BisGMA (%)	TEGDMA (%)	Filler Loading (wt%)
75	25	75
75	25	40
75	25	0

**Table 2:** pH-based storage media used for chemical ageing.

Storage Medium	Composition	pH
PBS	Dulbecco’s phosphate-buffered saline	7.2
Citric acid buffer	Citric acid + sodium phosphate dibasic	3.8
NaOH buffer	Sodium hydroxide + sodium bicarbonate	11

**Table 3:** Sorption and percentage mass increase following storage in PBS, citric acid, and NaOH. Values are median (IQR); sorption is expressed in μg/mm^3^.

	Filler	Sorption Day 1 (μg/mm^3^)	Sorption Day 28 (μg/mm^3^)	Mass Increase at Day 28 (MI%)
PBS	75%	2.3 (1.34)	5.5 (0.84)	1.2 (0.14)
	40%	2.9 (1.62)	7.7 (0.93)	2.1 (1.33)
	0%	5.3 (1.53)	10.5 (1.75)	3.8 (0.46)
Citric Acid	75%	3.0 (0.59)	5.6 (0.89)	1.1 (0.16)
	40%	3.6 (0.81)	6.3 (1.23)	1.8 (0.24)
	0%	3.7 (3.70)	8.6 (9.34)	3.5 (0.47)
NaOH	75%	3.0 (1.17)	3.5 (1.22)	0.7 (0.26)
	40%	4.0 (1.99)	8.4 (5.38)	2.0 (0.34)
	0%	6.6 (2.25)	10.3 (4.52)	3.5 (1.16)

**Table 4:** Vickers hardness at baseline, and after 1 and 28 days of storage. Values are median VHN (IQR).

Filler	Baseline	Storage Medium	Day 1	Day 28
75%	63 (14.0)	PBS	57 (10.9)	50 (19.7)
		Citric acid	56 (20.2)	45 (14.5)
		NaOH	27 (15.0)	48 (24.3)
40%	33 (13.4)	PBS	25.42 (2.8)	32 (24.2)
		Citric acid	27 (5.3)	29 (9.5)
		NaOH	21 (11.0)	28 (13.3)
0%	29 (4.4)	PBS	16 (12.6)	22 (8.5)
		Citric acid	23 (14.0)	27 (9.1)
		NaOH	25 (3.6)	19 (8.3)

**Table 5:** Nanoscale surface roughness (Sa, nm) following citric acid, NaOH, and PBS exposure. Values are median (IQR).

	Filler	Baseline	Day 1	Day 28
Citric acid	75%	112 (30)	117 (15)	97 (21)
	40%	126 (65)	176 (49)	113 (22)
	0%	186 (43)	257 (95)	274 (247)
NaOH	75%	112 (30)	165 (52)	110 (12)
	40%	126 (65)	109 (36)	129 (10)
	0%	186 (43)	213 (45)	297 (233)
PBS	75%	112 (30)	122 (25)	137 (73)
	40%	126 (65)	172 (37)	126 (28)
	0%	186 (43)	129 (58)	234 (49)

**Table 6:** Results summarise the observed changes in microscale and nanomechanical properties following pH-based ageing.

Property	PBS	Citric Acid	NaOH	Major Compositional Influence
Sorption/MI%	Mild	Mild	Mild	Strong effect of filler loading
Vickers hardness	Limited overall effect	Limited overall effect	Limited overall effect	Higher filler → higher VHN
SEM	Moderate degradation	Moderate degradation	Evidence of filler–matrix interfacial degradation	Filled materials showed interfacial effects
AFM topography	Limited obvious change	Limited obvious change	Limited obvious change	No consistent pattern
Sa roughness	Variable response	Variable response	Variable response	Higher filler loading was associated with lower Sa overall.
Young’s modulus	Delayed reduction	Shift towards lower values from Day 1	Shift towards lower values from Day 1	Early response across formulations

## Data Availability

The data supporting the findings of this study are available from the corresponding author upon reasonable request.
